# 4-Acetyl­pyridine–fumaric acid (2/1)

**DOI:** 10.1107/S1600536809020480

**Published:** 2009-06-06

**Authors:** Kan Xu, Bing-Yu Zhang, Jing-Jing Nie, Duan-Jun Xu

**Affiliations:** a Department of Orthopaedics, Second Affiliated Hospital, School of Medicine, Zhejiang University, People’s Republic of China; b Department of Chemistry, Zhejiang University, People’s Republic of China

## Abstract

In the crystal structure of the title cocrystal, 2C_7_H_7_NO·C_4_H_4_O_4_, the complete fumaric acid mol­ecule is generated by a crystallographic inversion centre. The two components of the cocrystal are linked by an O—H⋯N hydrogen bond.

## Related literature

For biological and medicinal applications of 4-acetyl­pyridine and fumaric acid, see: Fidler *et al.* (2003 ▶); Thomas *et al.* (2007 ▶). For mol­ecular complexes of neutral pyridine derivatives and neutral fumaric acid, see: Bowes *et al.* (2003 ▶); Aakeroy *et al.* (2002 ▶, 2006 ▶, 2007 ▶); Haynes *et al.* (2006 ▶); Bu *et al.* (2007 ▶). For literature on C—O bond distances in fumaric acid, see: Liu *et al.* (2003 ▶). For metal complexes of 4-acetyl­pyridine, see: Steffen & Palenik (1977 ▶); Pang *et al.* (1994 ▶). For a 4-acetyl­pyridinium salt, see: Kochel (2005 ▶).
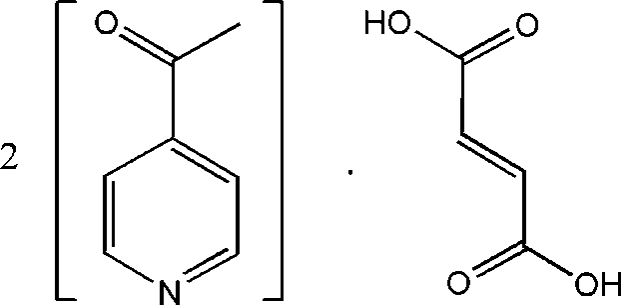


## Experimental

### 

#### Crystal data

2C_7_H_7_NO·C_4_H_4_O_4_
*M* *_r_* = 358.34
Triclinic, 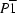

*a* = 3.9062 (5) Å
*b* = 8.6809 (13) Å
*c* = 13.0909 (18) Å
α = 87.925 (4)°
β = 89.941 (3)°
γ = 83.141 (4)°
*V* = 440.44 (11) Å^3^
*Z* = 1
Mo *K*α radiation
μ = 0.10 mm^−1^
*T* = 294 K
0.30 × 0.11 × 0.08 mm

#### Data collection

Rigaku R-AXIS RAPID IP diffractometer
Absorption correction: none
3600 measured reflections
1589 independent reflections
798 reflections with *I* > 2σ(*I*)
*R*_int_ = 0.030

#### Refinement

*R*[*F* ^2^ > 2σ(*F* ^2^)] = 0.040
*wR*(*F* ^2^) = 0.143
*S* = 1.18
1589 reflections
124 parameters
H atoms treated by a mixture of independent and constrained refinement
Δρ_max_ = 0.19 e Å^−3^
Δρ_min_ = −0.20 e Å^−3^

### 

Data collection: *PROCESS-AUTO* (Rigaku, 1998 ▶); cell refinement: *PROCESS-AUTO*; data reduction: *CrystalStructure* (Rigaku/MSC, 2002 ▶); program(s) used to solve structure: *SIR92* (Altomare *et al.*, 1993 ▶); program(s) used to refine structure: *SHELXL97* (Sheldrick, 2008 ▶); molecular graphics: *ORTEP-3 for Windows* (Farrugia, 1997 ▶); software used to prepare material for publication: *WinGX* (Farrugia, 1999 ▶).

## Supplementary Material

Crystal structure: contains datablocks I, global. DOI: 10.1107/S1600536809020480/ng2588sup1.cif

Structure factors: contains datablocks I. DOI: 10.1107/S1600536809020480/ng2588Isup2.hkl

Additional supplementary materials:  crystallographic information; 3D view; checkCIF report

## Figures and Tables

**Table 1:** Hydrogen-bond geometry (Å, °)

*D*—H⋯*A*	*D*—H	H⋯*A*	*D*⋯*A*	*D*—H⋯*A*
O3—H3*A*⋯N1	0.98 (4)	1.64 (4)	2.599 (3)	166 (4)

**Fig. 1.**
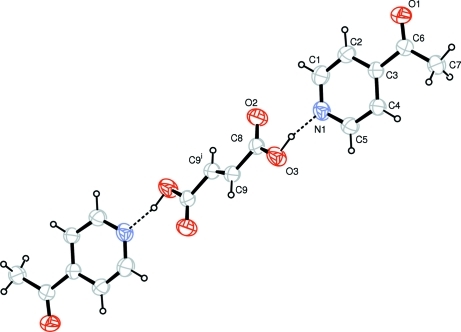
The molecular structure of the title compound with 40% probability displacement (arbitrary spheres for H atoms). Dashed lines indicate hydrogen bonding.

## supplementary crystallographic information

### Comment

The fumaric acid and acetylpyridine have been widely used in the biological and medicine fileds (Thomas *et al.* 2007; Fidler *et al.* 2003). In the medicine composition the carboxyl group of the fumaric acid is usually deprotonated while the pyridine derivatives are protonated. But some crystal structure determinations showed the neutral pyridine derivatives and fumaric acid in the crystal structures, *i.e.* the pyridine derivatives are not protonated while the fumaric acid is also not deprotonated in these crystal structures (Bowes *et al.* 2003; Aakeroy *et al.*, 2002, 2006, 2007; Haynes *et al.* 2006; Bu *et al.* 2007). Herein we report the crystal structure of the new compound containing pyridine derivative and fumaric acid components.

The crystal structure of the title compond consists of fumaric acid and 4-acetylpyridine molecules (Fig. 1). The planar fumaric acid molecule is centrosymmetric with the mid-point of the C═C double bond located at an inversion center. The C8—O2 bond distance of 1.204 (3) Å is much shorter than the C8—O3 bond distance of 1.297 (3) Å, it suggests that the carboxyl group is not deprotonated in the crystal structure (Liu *et al.* 2003).

The acetylpyridine molecule is not protonated in the crystal structure, which contrasts with that found in the crystal structure of the 4-acetylpyridinium chloride (Kochel, 2005). The geometry data of the acetylpyridine is consistent with those found in metal complexes of acetylpyridine (Steffen & Palenik, 1977; Pang *et al.*, 1994). The planar acetylpyridine molecule is twisted to the fumaric acid with a dihedral angle of 25.97 (11)° in the crystal structure.

The intermolecular classic O—H···N hydrogen bonding and weak C—H···O hydrogen bonding help to stabilize the crystal structure (Table 1).

### Experimental

Reagents and solvent were used as purchased without further purification. 4-Acetylpyridine (2 mmol) and fumaric acid (1 mmol) were dissolved in water–ethanol (6 ml, 1:5) at room temperature. The single crystals were obtained from the solution after 3 d.

### Refinement

The carboxyl H atom was located in a difference Fourier map and refined isotropically. Methyl H atoms were placed in calculated positions with C—H = 0.96 Å and the torsion angle was refined to fit the electron density, *U*_iso_(H) = 1.5*U*_eq_(C). Other H atoms were placed in calculated positions with C—H = 0.93 Å and refined in riding mode with *U*_iso_(H) = 1.2*U*_eq_(C).

### Crystal data

**Table:**

2C_7_H_7_NO·C_4_H_4_O_4_	*Z* = 1
*M**_r_* = 358.34	*F*(000) = 188
Triclinic, *P*1	*D*_x_ = 1.351 Mg m^−^^3^
Hall symbol: -P 1	Mo *K*α radiation, λ = 0.71073 Å
*a* = 3.9062 (5) Å	Cell parameters from 2308 reflections
*b* = 8.6809 (13) Å	θ = 3.2–24.6°
*c* = 13.0909 (18) Å	µ = 0.10 mm^−^^1^
α = 87.925 (4)°	*T* = 294 K
β = 89.941 (3)°	Needle, colourless
γ = 83.141 (4)°	0.30 × 0.11 × 0.08 mm
*V* = 440.44 (11) Å^3^	

### Data collection

**Table:**

Rigaku R-AXIS RAPID IP diffractometer	798 reflections with *I* > 2σ(*I*)
Radiation source: fine-focus sealed tube	*R*_int_ = 0.030
graphite	θ_max_ = 25.2°, θ_min_ = 3.1°
ω scans	*h* = −4→4
3600 measured reflections	*k* = −10→10
1589 independent reflections	*l* = −15→15

### Refinement

**Table:**

Refinement on *F*^2^	Secondary atom site location: difference Fourier map
Least-squares matrix: full	Hydrogen site location: inferred from neighbouring sites
*R*[*F*^2^ > 2σ(*F*^2^)] = 0.040	H atoms treated by a mixture of independent and constrained refinement
*wR*(*F*^2^) = 0.143	*w* = 1/[σ^2^(*F*_o_^2^) + (0.0527*P*)^2^ + 0.048*P*] where *P* = (*F*_o_^2^ + 2*F*_c_^2^)/3
*S* = 1.18	(Δ/σ)_max_ = 0.001
1589 reflections	Δρ_max_ = 0.19 e Å^−^^3^
124 parameters	Δρ_min_ = −0.20 e Å^−^^3^
0 restraints	Extinction correction: *SHELXL97* (Sheldrick, 2008), Fc^*^=kFc[1+0.001xFc^2^λ^3^/sin(2θ)]^-1/4^
Primary atom site location: structure-invariant direct methods	Extinction coefficient: 0.032 (8)

### Special details

**Table:**

Geometry. All e.s.d.'s (except the e.s.d. in the dihedral angle between two l.s. planes) are estimated using the full covariance matrix. The cell e.s.d.'s are taken into account individually in the estimation of e.s.d.'s in distances, angles and torsion angles; correlations between e.s.d.'s in cell parameters are only used when they are defined by crystal symmetry. An approximate (isotropic) treatment of cell e.s.d.'s is used for estimating e.s.d.'s involving l.s. planes.
Refinement. Refinement of *F*^2^ against ALL reflections. The weighted *R*-factor *wR* and goodness of fit *S* are based on *F*^2^, conventional *R*-factors *R* are based on *F*, with *F* set to zero for negative *F*^2^. The threshold expression of *F*^2^ > σ(*F*^2^) is used only for calculating *R*-factors(gt) *etc*. and is not relevant to the choice of reflections for refinement. *R*-factors based on *F*^2^ are statistically about twice as large as those based on *F*, and *R*- factors based on ALL data will be even larger.

### Fractional atomic coordinates and isotropic or equivalent isotropic displacement parameters (Å^2^)

**Table:**

	*x*	*y*	*z*	*U*_iso_*/*U*_eq_	
N1	0.4491 (6)	0.4588 (3)	0.69775 (18)	0.0630 (7)	
O1	0.8832 (6)	0.7499 (3)	0.98158 (16)	0.0900 (8)	
O2	0.1660 (6)	0.1037 (2)	0.67034 (15)	0.0775 (7)	
O3	0.2584 (6)	0.2870 (3)	0.55473 (16)	0.0764 (7)	
C1	0.5832 (8)	0.4178 (4)	0.7899 (2)	0.0733 (9)	
H1	0.6122	0.3130	0.8097	0.088*	
C2	0.6807 (7)	0.5230 (3)	0.8573 (2)	0.0651 (9)	
H2	0.7765	0.4894	0.9204	0.078*	
C3	0.6342 (6)	0.6786 (3)	0.82954 (19)	0.0494 (7)	
C4	0.4891 (6)	0.7228 (3)	0.73526 (19)	0.0546 (7)	
H4	0.4508	0.8270	0.7145	0.065*	
C5	0.4019 (7)	0.6088 (4)	0.6724 (2)	0.0612 (8)	
H5	0.3048	0.6391	0.6089	0.073*	
C6	0.7453 (7)	0.7954 (3)	0.9018 (2)	0.0562 (8)	
C7	0.6902 (7)	0.9624 (3)	0.8712 (2)	0.0653 (9)	
H7A	0.7914	1.0212	0.9215	0.098*	
H7B	0.4474	0.9961	0.8664	0.098*	
H7C	0.7961	0.9784	0.8061	0.098*	
C8	0.1658 (7)	0.1535 (3)	0.5833 (2)	0.0539 (7)	
C9	0.0643 (7)	0.0651 (3)	0.4951 (2)	0.0561 (8)	
H9	0.0951	0.1057	0.4295	0.067*	
H3A	0.335 (9)	0.337 (5)	0.615 (3)	0.131 (14)*	

### Atomic displacement parameters (Å^2^)

**Table:**

	*U*^11^	*U*^22^	*U*^33^	*U*^12^	*U*^13^	*U*^23^
N1	0.0771 (16)	0.0500 (17)	0.0641 (16)	−0.0142 (12)	−0.0009 (13)	−0.0083 (13)
O1	0.1244 (18)	0.0731 (17)	0.0703 (14)	−0.0004 (13)	−0.0407 (14)	−0.0077 (12)
O2	0.1167 (18)	0.0640 (15)	0.0548 (13)	−0.0234 (12)	−0.0138 (12)	0.0004 (11)
O3	0.1198 (18)	0.0541 (15)	0.0607 (13)	−0.0313 (13)	−0.0048 (12)	−0.0076 (11)
C1	0.096 (2)	0.046 (2)	0.077 (2)	−0.0096 (17)	−0.0064 (19)	0.0019 (17)
C2	0.082 (2)	0.052 (2)	0.0610 (19)	−0.0053 (15)	−0.0123 (16)	0.0030 (15)
C3	0.0523 (15)	0.0448 (18)	0.0513 (16)	−0.0058 (12)	−0.0004 (13)	−0.0030 (13)
C4	0.0682 (17)	0.0451 (17)	0.0512 (16)	−0.0100 (13)	−0.0046 (14)	−0.0014 (13)
C5	0.0704 (18)	0.057 (2)	0.0566 (18)	−0.0100 (15)	−0.0086 (15)	−0.0024 (15)
C6	0.0577 (16)	0.057 (2)	0.0541 (18)	−0.0048 (13)	−0.0047 (14)	−0.0051 (14)
C7	0.0738 (19)	0.054 (2)	0.070 (2)	−0.0163 (15)	−0.0083 (16)	−0.0077 (16)
C8	0.0604 (16)	0.0446 (18)	0.0567 (18)	−0.0048 (13)	−0.0084 (14)	−0.0063 (14)
C9	0.0680 (17)	0.0469 (18)	0.0534 (16)	−0.0063 (13)	−0.0072 (14)	−0.0016 (14)

### Geometric parameters (Å, °)

**Table:**

N1—C5	1.323 (4)	C3—C6	1.512 (4)
N1—C1	1.335 (4)	C4—C5	1.383 (4)
O1—C6	1.207 (3)	C4—H4	0.9300
O2—C8	1.204 (3)	C5—H5	0.9300
O3—C8	1.297 (3)	C6—C7	1.481 (4)
O3—H3A	0.97 (4)	C7—H7A	0.9600
C1—C2	1.378 (4)	C7—H7B	0.9600
C1—H1	0.9300	C7—H7C	0.9600
C2—C3	1.377 (4)	C8—C9	1.488 (4)
C2—H2	0.9300	C9—C9^i^	1.293 (5)
C3—C4	1.381 (3)	C9—H9	0.9300
			
C5—N1—C1	117.3 (3)	C4—C5—H5	118.2
C8—O3—H3A	108 (2)	O1—C6—C7	121.8 (3)
N1—C1—C2	123.2 (3)	O1—C6—C3	119.3 (3)
N1—C1—H1	118.4	C7—C6—C3	118.9 (2)
C2—C1—H1	118.4	C6—C7—H7A	109.5
C3—C2—C1	118.9 (3)	C6—C7—H7B	109.5
C3—C2—H2	120.5	H7A—C7—H7B	109.5
C1—C2—H2	120.5	C6—C7—H7C	109.5
C2—C3—C4	118.4 (2)	H7A—C7—H7C	109.5
C2—C3—C6	119.6 (2)	H7B—C7—H7C	109.5
C4—C3—C6	122.0 (3)	O2—C8—O3	124.8 (3)
C3—C4—C5	118.6 (3)	O2—C8—C9	123.1 (3)
C3—C4—H4	120.7	O3—C8—C9	112.2 (3)
C5—C4—H4	120.7	C9^i^—C9—C8	123.6 (3)
N1—C5—C4	123.5 (3)	C9^i^—C9—H9	118.2
N1—C5—H5	118.2	C8—C9—H9	118.2

Symmetry codes: (i) −*x*, −*y*, −*z*+1.

### Hydrogen-bond geometry (Å, °)

**Table:**

*D*—H···*A*	*D*—H	H···*A*	*D*···*A*	*D*—H···*A*
O3—H3A···N1	0.98 (4)	1.64 (4)	2.599 (3)	166 (4)
C4—H4···O2^ii^	0.93	2.57	3.471 (3)	164
C7—H7C···O2^iii^	0.96	2.58	3.489 (3)	158

Symmetry codes: (ii) *x*, *y*+1, *z*; (iii) *x*+1, *y*+1, *z*.
